# Teaching Disruption by COVID-19: Burnout, Isolation, and Sense of Belonging in Accounting Tutors in E-Learning and B-Learning

**DOI:** 10.3390/ijerph181910339

**Published:** 2021-09-30

**Authors:** Laura Parte, Teresa Herrador-Alcaide

**Affiliations:** Department of Business and Accounting, Faculty of Economics and Business Administration, Universidad Nacional de Educación a Distancia (UNED), Paseo Senda del Rey, 11, 28040 Madrid, Spain; therrador@cee.uned.es

**Keywords:** teacher burnout, feelings of isolation, sense of belonging, b-learning, e-learning, COVID-19 pandemic, accounting, higher education

## Abstract

This study examines burnout syndrome, feelings of isolation, and sense of belonging in a sample of accounting tutors enrolled in e-learning and b-learning modalities before and after COVID-19 disruption. The study also includes several sociodemographic and labour variables to better understand the three dimensions. The participants were tutors enrolled in two accounting courses at higher education during the academic years 2019–2020 and 2020–2021. Our results do not show high levels of tutor burnout syndrome, neither before COVID-19 disruption nor after COVID-19 disruption. Findings also reveal that the isolation perception of accounting tutors is not high in both periods, while the sense of belonging of the teaching community is high in both periods. The evidence also suggests some variations in dimension scores according to sociodemographic and labour variables, but the evidence should be interpreted with caution due to the sample size. Despite this limitation, to the best of our knowledge, this is the first study that evaluates burnout, feelings of isolation, and sense of belonging in a tutor collective in e-learning and b-learning before and after COVID-19 disruption.

## 1. Introduction

The COVID-19 epidemic put the online learning system under stress worldwide. The consequences of the global outbreak of COVID-19, including the switch from face-to-face classes to online classes at least during strict confinement (lockdown) and the acceleration of digitalization, has substantially increased the number of publications focused on e-learning, including the attitudes towards the unexpected change, teachers’ stress, and emotional problems, among others. Hence, there are still calls for more research focused on e-learning and b-blended modalities in higher education considering the expected growth for the coming years [1]. Scientific mapping on management and administration related to COVID-19 has shown that the trend of publications revolves around specific groups and the field of health, so making progress in other professional collectives can enrich scientific knowledge [2].

This paper examines several stressor factors associated with the teacher profession, such as burnout syndrome and feelings of isolation, in conjunction with the sense of belonging in b-learning and e-learning modalities, before and after COVID-19 disruption. In addition, we explore the relationship among dimensions and the effect of sociodemographic and labour variables.

Teaching has been socially considered a stressful profession, in all education modalities. Burnout refers to feelings, such as work being unsatisfactory, unpleasant, or unrewarding. Teacher burnout is a crucial problem because it is linked to disengagement in the classroom and disconnection with institutional goals, including those affecting the student and the overall success of the institution [3]. Méndez et al. [4] documented that teachers with high levels of burnout have high levels of depression and lower scores in self-esteem. At the university level, it is even more worrying because the university goes beyond education, being a dynamic agent for local and national development [5]. Therefore, understanding the factors associated with teacher burnout is not only relevant to foster job satisfaction, self-health, and learning outputs but also for the whole society.

According to Maslach and Jackson [6] and Maslach et al. [7], burnout as a syndrome has three dimensions: (1) emotional exhaustion that refers to feelings of being emotionally overextended and exhausted by one’s work, (2) depersonalization that is a feeling of impersonal response toward students, and (3) a lack of personal accomplishment and a loss of personal self-efficacy. Job demands-resources is also a useful framework to explain the burnout dimensions.

Extended research has examined teacher burnout using the Maslach Burnout Inventory (MBI) for educators and job demands-resources. Prior literature has focused mainly on burnout definition, adjustments, prevention, and factors associated with burnout syndrome related to teachers (such as self-efficacy, self-esteem), students (such as relatedness), organization, and environmental (for example, attitudes towards inclusion and organization support) and socio-demographic factors (such as gender, age, and work experience) [4,8,9,10,11,12,13]. More recently scholars have paid special attention to teacher burnout during the COVID-19 pandemic, mainly examining the switch from face-to-face classes to the online setting [14,15,16,17].

However, little research has been conducted in higher education compared to other professions, and also in other stages of education (3), and there is still room [11]. Furthermore, there is little evidence in the blended university and distance university compared to traditional education [18,19]. Distance education is an interesting case study due to its differences from traditional education. E-learning is characterized by an asynchronous learning environment, rapid technology changes, time management, perceptions of isolation, learners’ diversity, and multicultural factors and complexities, among others. The growing academic research on e-learning during strict confinement and also in the post-COVID-19 era makes this study valuable.

Building directly upon the first objective of this research, another stressor variable in the e-learning modality is the isolation perception of members of the community. Isolation is associated with a perception of loneliness that members experience in the learning-teaching process. In the teachers’ collective, isolation is linked to an absence of communication between peers [20] and an absence of support by peers [21], which essentially engage in a sense of not being part of a community [22], and can cause cognitive distortions [23] and mental health problems [24]. Online teaching modalities have also associated with a feeling of being academically alone. The isolation perception after the COVID-19 disruption is an interesting research question as some studies have found that the disruption resulted in positive attitudes toward distance education [25].

In addition, the sense of belonging to the academic community and professional identity have attracted the attention of researchers [14,26]. This study also examined the sense of belonging in e-learning and b-learning modalities before and after COVID-19 disruption. According to the social cognitive career theory, the realization of an individual career depends mainly on three factors: belief, process, and motivation. Chen et al. [14] argue that belief can be proxied by professional identity, the process is job satisfaction, and the motivation is job burnout. It is presumed that teachers with a greater sense of professional identity are less likely to experience job syndrome.

The collective of tutors and e-tutors have rarely been examined in higher education, even in distance education, despite playing a fundamental role in e-learning and b-learning modalities. In general, in the e-learning modality, tutors are responsible for facilitating online delivery of tutorial material, students’ interactions with their peers online, and students’ learning experiences online, among others. In the b-learning modality, tutors also teach face to face. In both modalities, tutors must be a learning manager, by facilitating students’ acquisition of skills that allow them to “learn to learn”. Learners’ heterogeneity and diversity in the e-learning and b-blended modality make the tutors’ function complex compared to other education contexts. The tutor assumes high levels of responsibility, which can cause stress and lead to burnout syndrome. Considering the tutor’s role in the distance education model, it is of special interest to describe the status of burnout as well as other feelings of isolation and the sense of belonging.

The recent global pandemic has forced a switch from face-to-face teaching, including b-learning, towards the e-learning modality from one day to the next around the world. Garvey et al. [27] found that around 89.9% of students in a traditional university suffered from abnormal anxiety during the strict confinement process in Spain. The strict confinement lasted several weeks, and after that, hybrid or e-learning models were combined intermittently in many countries. This situation led many tutors to adapt their teaching routines in a short period, having to further develop technological and didactic skills, and assuming even more of a learning manager role. The new scenario can increase teachers’ stress, burnout syndrome, and feelings of isolation, seriously affecting tutors’ performance. Nevertheless, the psychological effects, including stress, have hardly been analysed in the tutor collective in higher education, and even less than other collectives in the e-learning and b-learning modalities.

The current study examined this setting by selecting a sample of accounting tutors enrolled in two accounting courses offered by higher education during the academic years 2019–2020 and 2020–2021. Our participants taught in the first semester of the course. The 2019–2020 academic year is characterized by normal teaching (before the pandemic was declared in Spain and any sanitary measures establishing and, therefore, without alteration of the social and sanitary conditions of the country). On contrast, in the first semester of the 2020–2021 academic year, COVID-19 had already occurred, and the first lockdown had already occurred from March to June 2019. Thus, we measured the dimensions (burnout, isolation, and sense of belonging) prior to the COVID-19 disruption (first semester of the year 2019–2020) and after the COVID-19 disruption (first semester of the year 2020–2021).

Furthermore, it is noted that burnout, feelings of isolation, and sense of belonging around accounting and finance disciplines have been less explored compared to other subjects. In this point, we complement previous studies in the accounting and finance discipline [9,28] but include some progress in the line of research. First, there are calls for further research in different disciplines because pedagogical and andragogical variables can affect the results [18,29]. Second, we explored teachers’ stressor in e-learning and b-learning together, including the COVID-19 disruption. Finally, we explored a sample scarcely examined in the literature.

The structure of the paper is as follows. The second section deals with the literature review and the research questions. The third section explains the method and materials, including participants, dimensions, measures and instruments, survey procedure, and descriptive statistic. The fourth section presents the results. The fifth section discusses the results, provides some recommendations, explains the limitations, and offers suggestions for future results. The last section provides conclusions.

## 2. Literature and Background

### 2.1. Burnout Definition

Maslach et al. [7] explain that burnout is a syndrome of reduced personal accomplishment, increased emotional exhaustion, and increased depersonalization experienced by individuals working closely with people. Maslach and Leiter [30] (p. 103) consider that teacher burnout is “a psychological syndrome emerging as a prolonged response to chronic interpersonal stressors on the job”. In 2019, the World Health Organization (WHO) provide an updated definition of burnout considering that is “a syndrome conceptualized as resulting from chronic workplace stress that has not been successfully managed. It is characterized by three dimensions: feelings of energy depletion or exhaustion; increased mental distance from one’s job, or feelings of negativism or cynicism related to one’s job; and reduced professional efficacy” [31].

### 2.2. Burnout Syndrome in Higher Education

Teaching has been considered a stressful profession in several education modalities and societies. Pyhältö et al. [12] found that in Finland, which is considered one of the countries with the best educational model, nearly half of the teachers in their sample showed different combinations of burnout. Additionally, in Australia, nearly half of all teachers drop out of the profession within the first five years [32]. Abós et al. [33] found that certain work stressors negatively affect the basic psychological needs of teachers, which are essential to achieve optimal psychological functioning. Teacher burnout has consequences for the teachers’ collective, students’ collective, educational organizations, and society [3,12,13,34]. Therefore, understanding the factors associated with teacher burnout is not only relevant to foster job satisfaction, self-health, and the learning output, but also for the whole society.

Drawing on teacher burnout, several studies have used the MBI scale to understand teacher burnout and its dimensions. It is common to examine cognitive, attitudinal, organizational, environmental, teacher, and student variables. Normally, empirical studies deal with a set of factors related to potential burnout factors to understand the syndrome. The organization variables are broadly examined in the literature. Maslach and Jackson [35] pointed out that the primary reasons for burnout are workplace factors rather than the personal characteristics of employees. For example, Pyhältö et al. [34] used interviews to study teacher burnout in Finland. The results revealed that teacher burnout is mainly associated with unsolved problems in social interactions with students, parents, and within the professional community. Hence, the phenomenon is perceived as social, collective, and relational rather than only individual. In contrast, Klusmann et al. [29] suggested that most of the variability in teachers’ emotional and motivational is explained by individual factors rather than organizational variables. Therefore, more studies are needed to explore the individual characteristics between teachers. It has also been identified that an absence of teacher burnout is not directly connected with high levels of work engagement [29].

In the literature review provided by Watt and Roberson [3], it was noted that teacher burnout is strongly associated with high numbers of students, especially in postgraduate courses. Lowenstein [36] and Klusmann et al. [29] also argued that large class sizes are associated with teacher burnout while Saloviita and Pakarinen [13] found that class size has a marginal effect. Lowenstein [36] argued that teacher burnout is caused by several factors, such as an absence of social recognition of teachers, large class size, absence of resources, isolation, fear of violence, absence of classroom control, role ambiguity, limited promotional opportunities, and lack of support. Lau et al. [8] used MBI in a sample of teachers in Hong Kong and found that teachers who were younger, unmarried, less experienced, did not have religious beliefs, and still completing their professional trainee were more burned out.

Several papers have found that teachers’ self-efficacy is strongly associated with burnout. Specifically, teachers’ self-efficacy positively affects the degree of involvement, allowing teachers to deal with complex situations and cope with stress, and also positively affects student learning and student output [13]. The interaction between teachers and students is also a key variable. Scholars have shown that positive relationships with students can be create a good classroom environment and influence learning achievement [12,13,36]. Saloviita and Pakarinen [13] revealed that teachers’ efficacy, relatedness with students, perceptions of support, and attitudes towards inclusion are negatively associated with teacher burnout.

Regarding age and work experience, several authors have argued that older teachers have more work experience and generally well-known routines compared to their younger colleagues. Older teachers also know the profession and have more experience in dealing with the stress. Early career teachers can experience greater feelings of isolations, organizational pressures, and feelings of a lack of support. However, the empirical evidence is mixed. Some scholars have found that older teachers experience less burnout, especially the exhaustion dimension [3,8,36], but other scholars have found that older teachers are more exhausted and report less engagement [29]. Other scholars found that the age variable is not statistically significant, at least when the variable is continuous [13]. In this context, it is important that institutions and universities help early career teachers to adapt to the teaching profession and in facing burnout [8]. Regarding the gender variable, Watt and Roberson [3] argued that studies commonly show that males have a high score in the depersonalization dimension and females have a high score in the emotional exhaustion dimension and a high level of a lack of accomplishment [8,9]. Other studies have found that gender is a statistical marginal [13].

In the online setting, Hogan and McKnight [18] used an MSI-Educators survey on a sample of 76 online instructors and found that online instructors experience a high degree of depersonalization, low degree of personal accomplishment, and an average score on the emotional exhaustion subscale. They also detected that burnout syndrome in the online setting is lower than face-to-face classes (see [18]). In addition, they did not detect statistically significant differences in the gender variable, but females scored higher on all three burnout dimensions compared to male colleagues. They also pointed out that burnout in distance universities is in its infancy compared to traditional education.

Later, McCann and Holt [19] examined teacher burnout in online courses among higher education faculty members. The results showed that online instructors are less stressed than face-to-face teachers. The evidence also showed that the burnout dimensions: emotional exhaustion, depersonalization, and personal accomplishments, improved over time. It seems that the passage of time increases their expertise in the online modality, resulting in more familiar and comfortable teaching. Moreover, they did not find significant correlations between burnout and years of online work experience, gender, educational level, and academic training. These authors also called for more studies on online courses in higher education. An area of special interest in the online setting is how technology (educational technology, technological developments, technological pedagogical knowledge, changes in technology, etc.) influences teachers’ stress, considering mental health and burnout, among other symptoms [1].

COVID-19 has resulted in social isolation due to strict confinement for several months, followed by a period with severe mobility restrictions. Academia has paid special attention to the consequences of the COVID-19 pandemic on mental health, including stress, physical health impairment, and psychiatric disorders. The effects on the learning community—including all the agents implicated—are not an exception. Daumiller et al. [15] examined the shift from face-to-face to online teaching. The evidence showed that some instructors were successful but others had difficulties. Teachers’ learning approach goals and teachers’ attitudes were key variables associated with success. Hence, teachers’ avoidance of goals increased their burnout levels, resulting in worse student learning.

Sokal et al. [16] examined the first three months of the pandemic, using a sample of Canadian teachers. The evidence showed that teachers experienced an increase in some emotions, such as exhaustion and cynicism, but also an increase in efficacy in classroom management and a sense of accomplishment. It is also noted that teachers’ cognitive and emotional attitudes toward change were more negative. In a subsequent study, Sokal et al. [17] revealed that teacher demands are associated with the initial exhaustion stage of burnout, but further research is needed in the last stage because it is possible that the initial situation changes.

In addition, burnout in accounting and finance teachers has been less explored compared to other disciplines. Byrne et al. [28] used a sample of 100 accounting and finance academics in higher education and found that accounting and finance academics have low or average burnout considering emotional exhaustion and depersonalization but have a high degree of burnout considering the personal accomplishment dimension. Arquero and Donoso [9] used a sample of 192 Spanish teachers and found that variables, such as gender, time investment dedicated to research, recognition by students, grade of independence for decision making, and intention to drop-out, are associated with teacher burnout. Considering the growing relevance of research activities to promote the university, it could be reasonable that teacher burnout may be even worse in the coming years in teachers oriented towards research.

### 2.3. Feelings of Isolation at Distance University

Not far from burnout syndrome is the problem of teachers’ isolation. The feeling of isolation has been studied within the theory of social capital, considering the teaching approach from the professional perspective. Prior literature has focused on explaining how teaching is a profession with a high level of individualism, including the e-learning modality [37], especially as a modality of support. Teachers’ individualism has been studied in connection with professional isolation. This moves away from the idea of feeling part of a community, which generates a perceived feeling of isolation. The individualism is based not only on structures, but also on beliefs and practices, and departs from the ideal teaching profession, where social relationships and interactions between peers facilitate knowledge and information [38].

Teacher isolation has been studied using two main approaches. The first refers to the conditions in which teachers work and interact with their colleagues, and the second is related to personal psychological status rather than work conditions [39]. Several scholars have focused on explaining the factors associated with teacher isolation, including variables, such as a lack of or scarce interaction with classmates [20], absence of support by the teaching community [21,22,40], lack of a satisfaction in the social environment, and loss of a close attachment relationship [23].

Several scholars have suggested that feelings of isolation are more evident in higher education as universities are commonly organized in departments, which does not favour interdisciplinary discourse about how students are performing [41], combined with the problem of finding colleagues with the same degree of specialization to discuss academic contents [42], thus resulting in limited opportunities to discuss student learning. This also extends to the tutor collective. Interaction with other tutors to share impressions, interchange ideas, prepare learning material, etc. could be difficult. Moreover, they may not spend as much time at the university. Recent studies on distance universities highlight the importance of interaction between all members of the learning community to alleviate and prevent several problems, such as isolation and dropouts [43].

Traditionally, it was thought that teacher isolation is high in the online learning modality due to a lack of social interaction and lack of physical presence. Although online education has strong frameworks that allow cognitive, teaching, and social interaction, the introduction of ICT can lead to “unequal loneliness” [44]. Empirical studies have also shown that loneliness causes cognitive distortions and life dissatisfaction, and age acts as a moderating factor. Indeed, loneliness is associated with both psychological and sociological problems [23]. Currently, the new social isolation caused by the COVID-19 lockdown resulted in closures of colleges and universities worldwide. Extensive research has been developed to examine how teachers have coped with this unexpected situation. Most of them have found an increase in stress levels, emotional problems, anxiety levels, and symptoms of negative effects on mental health, among others. To a lesser extent, studies have reported teachers’ success in the new learning context, e.g., the literature provided by [1]. Nowadays, interaction among teachers could be even more necessary, due to the social distancing caused by the pandemic.

It is at this point that isolation is linked to burnout [39]. For example, Lowenstein [36] suggested that isolation, lack of support, absence of social recognition, large class size, fear of violence, absence of classroom control, role ambiguity, and limited promotional opportunities contribute to teacher burnout. Ortega-Jiménez et al. [24] documented that psychological stress, loneliness, and psychological inflexibility are related with poorer mental health and professional performance in a sample of 902 university teachers. As Pyhältö et al. [12] explained, several empirical studies have revealed that social interrelations can enhance teachers’ well-being, and, in particular, positive relationships with colleagues decrease teachers’ stress. Hence, UNESCO’s sustainable development includes well-being as a goal of individual and community development for sustainable growth and development [45]. The prosocial classroom model explains how teachers’ well-being and socioemotional functioning are crucial to manage the class and effectively lead educational instruction [11]. In fact, people with high levels of self-efficacy, optimism, hope, and resilience feel more satisfied can increase their skills to cope with burnout, thus leading to less burnout [10]. Then, it is important to make progress towards achieving high quality of life of people considering both ecological and socioeconomic environmental factors.

### 2.4. Sense of Belonging to the Academic Community at Distance University

Several scholars have also focused on professional identity in higher education. Beijaard et al. [26] divided prior studies into three approaches according to their objectives: teachers’ professional identity formation, identification of characteristics related to teachers’ professional identity, and professional identity in teachers. However, little research to date exists on the association between professional identity and job burnout among university teachers [14] and in the collective of tutors in e-learning and b-blended modalities. Considering the social cognitive career theory, professional identity plays a key role in teachers’ performance, because it can ward off feelings close to burnout syndrome. A sense of belonging in online teachers is even more necessary, because their social and professional isolation [46] can lead to feelings of loneliness as a barrier and other problematic feelings [47]. Specifically, teachers with high professional identity better understand their tasks, functions, and work; are more likely to accept the nature of the work; are able to adjust their expectations adequately; and show positive professional values [14]. Indeed, Pyhältö et al. [12] explained that several scholars have revealed that a poor sense of community and destructive friction in social interactions can lead to teacher burnout.

### 2.5. Purpose and Research Questions

Our objective was to examine burnout syndrome, feelings of isolation, and sense of belonging in accounting tutors in higher distance education, considering e-learning and b-learning modalities, in two periods: the pre- and post-COVID-19 scenario. Specifically, this study addressed eight research questions:

Do accounting tutors at higher distance educations suffer from burnout syndrome?

Do accounting tutors at higher distance educations perceive feelings of isolation?

Do accounting tutors at higher distance educations feel a sense of belonging to the teaching community?

Is there a relationship between burnout syndrome, feelings of isolation, and sense of belonging?

Do accounting tutors at higher distance educations perceive burnout syndrome differently from before to after COVID-19 disruption?

Do accounting tutors at higher distance educations perceive feelings of isolation differently from before to after COVID-19 disruption?

Do accounting tutors at higher distance educations feel a sense of belonging to the teaching community differently from before to after COVID-19 disruption?

**Figure 1:**
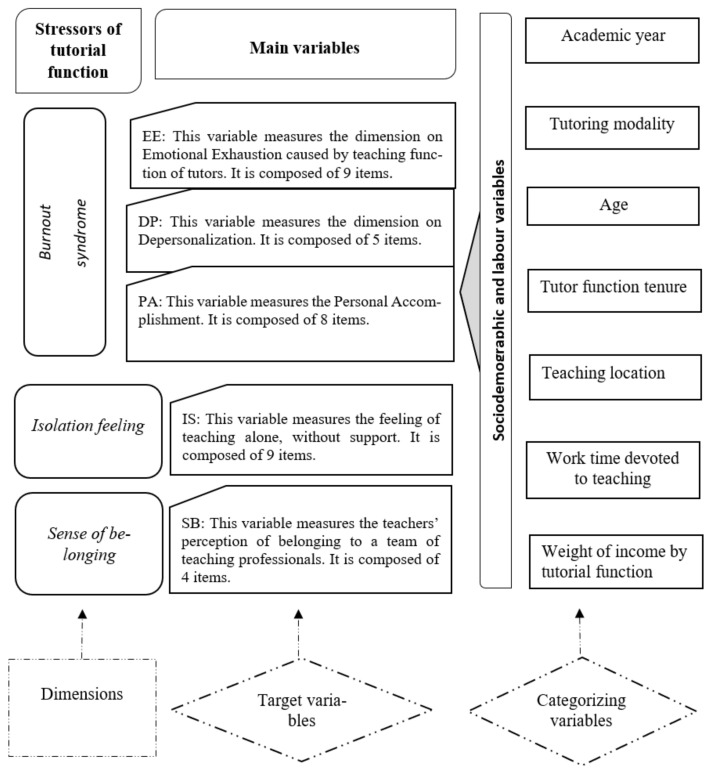
Study design. Source: Authors.

Therefore, the study addressed three main dimensions: Burnout syndrome, feelings of isolation, and sense of belonging. Each dimension is composed of several items or main variables. In addition, we included sociodemographic and labour variables as categorizing variables like age, tutoring modality, tutorial function tenure, work time devoted to university teaching, teaching location, and weight of income by tutorial function. Figure 1 summarises the design of the study.

## 3. Materials and Method

This section explains the materials, methods, and academic environment of the study. The first subsection explains the participants and tutorial framework. Later, the dimensions, variables, measures, and instruments are explained. The survey and procedure are also explained. The final subsection shows the descriptive statistics.

### 3.1. Participants and Tutorial Role Framework

The study was conducted at UNED (Universidad Nacional de Educación a Distancia). UNED was created in 1973 through the modality of distance education and it is the main hybrid and distance learning university in Spain. UNED is a large university in Europe, with a high volume of students. Another relevant characteristic is that students are generally part-time and mature.

The participants were tutors teaching accounting courses in the tourism degree during the academic years 2019–2020 and 2020–2021. Specifically, the tutors taught financial accounting and managerial accounting for tourism in the first semester of the academic year. Therefore, the period of analysis consisted of a pre-COVID-19 period (2019–2020) and a post-COVID period (2020–2021).

**Figure 2:**
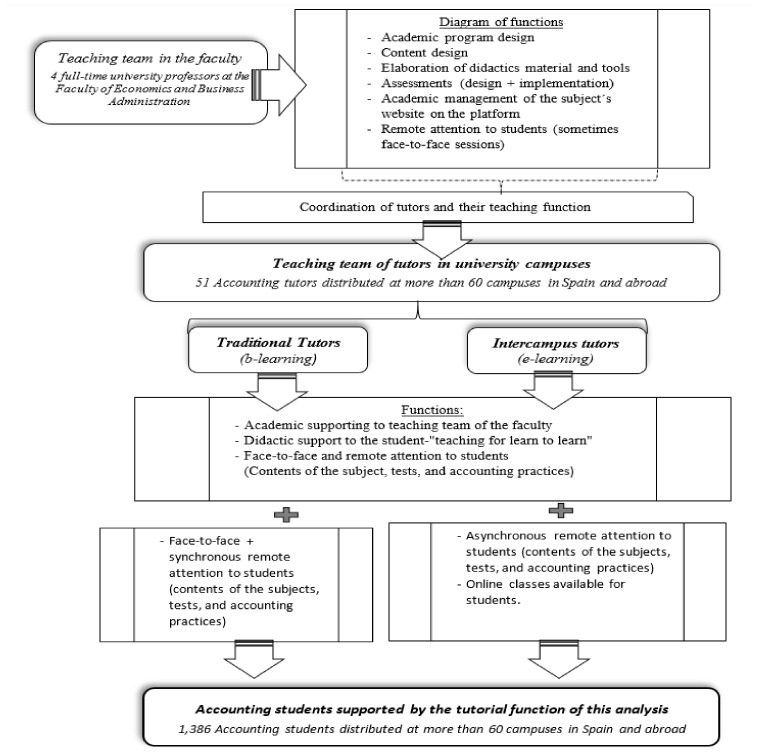
Educational model and tutor function in financial accounting and managerial accounting for tourism at UNED. Source: Authors.

There are two main tutorial functions: traditional tutorial function and intercampus tutorial function. The main differences are related to the role assumed for teaching, that is a learning manager with an online modality for intercampus modality and a learning manager that also supports face-to-face sessions for traditional tutors (see Figure 2). The learners’ heterogeneity and diversity in the e-learning and b-blended modality make the tutors’ function complex compared to other education contexts. The tutor assumes high levels of responsibility, which can cause stress and lead to burnout syndrome. They can also perceive feelings of isolation and low levels of a sense of belonging.

### 3.2. Dimensions, Variables, Measures, and Instruments

The teaching stressors were examined through three dimensions: Burnout, isolation, and sense of belonging. Based on previous educational studies, we used the Spanish translation of the MBI for Educators [9,48] with some adjustments to collect the tutor teaching and tutor function. The MBI assesses the level of burnout through three dimensions: emotional exhaustion (EE), depersonalization (DP), and personal accomplishment (PA). The MBI-ES consists of twenty-two statements rated on a Likert scale.

The EE subscale contains nine questions measuring overextension and exhaustion in the workplace. For example, “I feel emotionally exhausted by my tutoring”. The DP comprises five questions and measures impersonal responses toward coworkers and service recipients. For example, “I don’t really care what happens to some students”. The PA contains eight questions that measure feelings of competencies and success in the workplace, such as, “I have accomplished many worthwhile things in my tutoring”. A 5-point scale is used ranging from 1 to 5 [14,49].

A total score for each dimension is calculated as the sum of the response to the corresponding items. High scores on EE and DP and low scores on PA indicate a high level of burnout. Moderate scores on the three subscales indicate a moderate level of burnout. According to Maslach et al. [7], scores of the MBI subscales are considered high if they fall into the upper third portion of the normative distribution, average if they fall into the middle third, and low if they fall into the lower third.

Seisdedos [48] validated MBI for Spanish professions, showing the following levels: EE, 0.82; DP, 0.79; and PA, 0.79. However, for a teacher sample, the values were lower: EE, 0.83; DP, 0.48; and PA, 0.77. It was also noted that in the Spanish language, the Cronbach’s alpha is lower than in the English language and particularly low in the DP dimension [49].

The isolation dimension includes nine items related to feelings of isolation according to the previous literature [20,21,22,40] This subsection includes questions, such as, “The use of asynchronous communication, such as emails and messages, makes me feel isolated from the rest of my colleagues” and “The lack of physical presence of students makes me feel isolated from de rest of my colleagues”. In this section, we used a Likert scale (a 5-point scale) ranging from “strongly disagree” to “strongly agree” with a neutral midpoint. A total score is calculated as the sum of the response to the corresponding items. The higher the score, the stronger the feelings of isolation.

The sense of belonging includes four questions related to the feeling of being part of the teaching community [20,40]. The questions are as follows: “The relationship with other tutors makes me feel integrated into the teaching community” and “The relationship with the coordinator of the subject makes me feel part of the teaching community”. In this section, we used a Likert scale (a 5-point scale) ranging from “strongly disagree” to “strongly agree” with a neutral midpoint. A total score is calculated as the sum of the response to the corresponding items. The higher the score, the stronger the sense of belonging.

In this paper, we focused on the dimension scores (burnout, isolation, and sense of belonging) before and after COVID-19. In addition, we also calculated a set of statistical tests to examine if there are differences between the means according to the target variable. The null hypothesis proposes an equality of means (H_0_) for the dimensions before and after COVID-19 disruption while the alternative hypothesis (H_1_) establishes that the dimension means are not the same before and after COVID-19 outbreak. If the *p*-value is less than the cut-off, the null hypothesis is rejected. If the *p*-value is higher than the cut-off, the null hypothesis fails to be rejected. We also used power tests for the effect size: d-Cohen. The d-Cohen is a standardized mean difference and is commonly used to measure the effect size. It helps to measure how large an effect is. When the effect size is large, the statistical power increases [50,51]. The power test of d-Cohen is interpreted as a middle effect for the interval 0.20 < *d* ≤ 0.50 [52]. Although it is difficult to detect statistically significant differences in a small size sample, we also used non-parametric tests (U-Mann Whitney and Kruskal-Wallis tests) to test the differences in medians according to the target variable. However, as we commented through the paper, the statistical tests should be interpreted with caution due to the sample size.

The study also included socio-demographic and labour variables commonly used in prior educational literature and distance education modality, such as gender, age, tutoring modality, tutorial function tenure, work time devoted to university teaching, teaching location, and weight of income by tutorial function. The existing literature shows how sociodemographic and work characteristics moderate perceptions about burnout, isolation, and sense of belonging to the community. We used the same set of statistical tests, but again, we highlight that the results should be considered with caution.

### 3.3. Survey and Procedure

We administrated a survey with three key dimensions: MBI-ES questions, isolated questions, and sense of belonging questions. The survey also contained socio-demographic and labour variables.

The survey was administrated online. The person responsible for the course (coordinator) sent an email to all the tutors enrolled in two accounting courses in the first semester with a cover letter explaining the objectives of the project. An attachment with each email contained the survey with all the sections. The class tutoring in the first semester ended in January. We sent the survey three weeks before the end of the semester. We also sent two reminders to the tutors. The tutors voluntarily attended the survey. The answers were treated anonymously as we mentioned in the cover letter. In total, 28 tutors completed the survey. The response rate was 55%. The data were examined using the IBM SPSS Statistics 25 (IBM, Armonk, NY, USA).

### 3.4. Descriptive Statistics

Table 1 shows the descriptive statistics. The percentage of female tutors was 35.71% and the percentage of male tutors was 64.29%. With respect to teaching modality, 75.00% taught face to face with an AVIP class and 25.00% taught in the intercampus modality. In terms of age, 7.14% of the participants were 20–25, 21.43 were 26–35, 46.43% were 36–45, and 25% were 46–50 years. Regarding teaching experience, 35.71% had 0–5 years, 7.14% had 6–10 years, 32.14% had 11–20 years, and 25% had over 20 years of experience.

Most tutors indicated that they have family responsibilities (82.14%) and a part-time contract (75%). Specifically, they compatibilized tutoring at UNED with another job. In terms of tutoring activity or locality, 57.14% taught in the same locality and 42.86% moved to another locality for teaching. Regarding the weight of salary by tutorial function, for 76.92%, it represented less than 20%; for 15.38%, it represented between 21% and 40%; and for 7.69%, it represented more than 80% (see Table 1).

## 4. Results

This section is organized in several subsections. First, we show the MBI dimensions, IS dimension, and SB dimension. Second, we examine the correlation between the dimensions of the study. Third, we compare the dimensions of the study before and after COVID-19 disruption. In the last subsection, we show the dimensions according to the sociodemographic and labour variables. We also compare the results with prior evidence in the field.

### 4.1. Analysis of MBI, IS, and SB Dimensions

The Cronbach’s alpha takes values of EE, 0.70; DP, 0.45; PA, 0.74; IS: 0.83; and SB, 0.76. The Cronbach’s alpha takes values similar to other Spanish studies, e.g., [48,49]. It is also noted that prior papers validated Cronbach’s alpha above the normal value considered as the reference point when the number of items contained less than 10 items, e.g., [53].

Table 2 shows the scores of burnout, IS, and SB. The dimension averages of the burnout score are 13.75 (EE), 7.96 (DP), and 31.89 (PA). According to Maslach et al. [7], the scores of the MBI subscales are considered high if they fall into the upper third portion of the normative distribution, average if they fall into the middle third, and low if they fall into the lower third. Thus, our results show values located in the lower third of the scale for all the EE and DP subscales, and in the upper third in the PA, which implies low burnout syndrome. The correlation analysis shows a positive correlation between EE and DP and a negative correlation between EE and PA and also between DP and PA. The correlations are statistically significant (*p* < 0.05).

Arquero and Donoso [9], according to the rule of the three portions of Maslach et al. [7], positioned EE and DP in the lowest third of the score, and PA in the highest, in a sample of accounting teachers in the face-to-face modality in higher education in Spain in the group oriented towards teaching activity. Similar results were obtained by McCann and Holt (19) in a sample of 65 online university instructors in the United States. However, Hogan and McKnight [18] found that EE is in the middle third, DP in the highest, and PA in the lowest using a sample of 78 online instructors in higher education in the United States. The last two studies found high standard deviations compared to our sample.

Regarding the isolation dimension, our results did not show high levels. Indeed, most of the responses were over 75%, showing that tutors disagree with the perception of feelings of isolation when teaching in an online setting. In contrast, the sense of belonging was high. Most of the responses were over 75%, revealing that tutors agree with the perception of being part of the teaching community.

Several previous papers found that feelings of isolation are related to the perception of not being part of the teaching community or a lack of communication between teachers [20], but only a third of our tutors identified these causes as reasons for isolation. Unlike other authors, our tutors did not consider that large groups of students are linked to feelings of isolation [21,22,40] or contribute to teachers’ stress [3,36]. The results can be interpreted according to the learning modality. In general, both synchronous and asynchronous modalities support groups with more students compared to traditional education.

### 4.2. Correlations among Dimensions

Table 3 shows the correlation analysis among dimensions. Accordingly, the results show that there is a significant positive correlation between burnout and IS (*p* < 0.1) and a significant negative correlation between IS and SB (*p* < 0.05). Nevertheless, the negative correlation between burnout and SB is not statistically significant.

### 4.3. Analysis of the Dimensions before and after COVID-19 Disruption

Table 4 shows that the burnout dimensions and SB dimension were slightly worse after the COVID-19 disruption. The levels of EE, DP, and SB increased slightly, while the levels of PA, IS, and SB were slightly lower after the COVID-19 disruption. Although the sample size is small and the statistical tests need to be interpreted with caution, we calculated the t-test and non-parametric test between the pre-COVID-19 period and the post-COVID-19 period. All tests showed no significant differences between both periods (*p* < 0.05). In addition, d-Cohen showed a middle effect for DP (−0.27), IS (0.20) and SB (0.41).

Many scholars have found an increase in some dimensions and emotions associated with teachers’ stress during the strict confinement or in the months after strict confinement [16,17]. Our evidence does not show high increases in burnout dimensions. It is important to interpret the results within the setting. The distance learning modality experienced less change compared to traditional education because the system was already prepared for an online modality. Indeed, feelings of isolation decreased slightly because the the online modality supports cognitive, teaching, and social interaction among the participants. However, we cannot ignore the burnout dimensions being slightly worse.

### 4.4. Analysis of the Dimensions According to the Sample Characteristics

Table 5 reveals that the EE score is slightly higher for face-to-face teaching in AVIP classes (traditional tutors) compared to the intercampus modality. The former requires face-to-face teaching with students using the AVIP class, while the intercampus modality allows the option to record the class in the private modality without face-to-face teaching. If the tutor chooses the private modality, it is logical to experience a lower EE score as the tutor pressure decreases because he (she) can repeat the class several times. In the intercampus modality, the tutor experiences high levels of the DP score and low levels of PA. Specifically, the e-learning context faces greater levels of DP and low levels of PA compared to the blended modality. Although we found differences in the burnout dimension scores between both modalities, the t-test and non-parametric test were not statistically significant (*p* > 0.05). As we mentioned before, the statistical tests need to be interpreted with caution due to the sample size. The IS and SB dimensions are similar in both teaching modalities. The t-test and non-parametric test were not statistically significant (*p* > 0.05). The power test measured by d-Cohen showed a middle effect (0.20 < d ≤ 0.50) for EE (0.45), DP (−0.49), PA (0.30), and SB (−0.38).

Table 6 and Table 7 show that EE, PA, and IS, were worse in older and more experienced tutors compared to less mature and less experienced tutors. The results could be explained by the rapid technology change in distance education and its mandatory adoption for all teachers, due to the increasing weight that both teaching and online procedures are having in the educational model at UNED. It should be considered that in its founding, the distance educational model at UNED was a pioneer in some resources, such as TV and radio. Regarding the SB dimension, the score was higher in less mature tutors compared to more mature tutors. The dimension DP was also high in younger and less mature tutors. It is possible that older and more mature tutors with a high technology gap feel a lesser sense of belonging compared to younger tutors who are more technologically prepared, and usually younger teachers are more committed to online meetings and interaction. 

Considering the H-Kruskal Wallis test among the age intervals (25–45, 46–55, and 56–65), the dimension EE was statistically significant (*p* < 0.05), while the dimensions DP, PA, IS, and SB were not statistically significant (*p* > 0.05). However, we cannot discard the useful information driven by the average scores among age groups. Regarding the H-Kruskal Wallis test among work experience or tutorial tenure (0–5, 6–20, +20), the test did not show statistically significant differences (*p* > 0.05), but the small size makes it difficult to interpret the statistical tests. Similarly, the average dimension scores allow us to observe some interesting results among tutorial tenure, although the statistical tests did not achieve the levels of significance required (*p* < 0.05).

Prior literature has found mixed results regarding age and work experience. Some empirical papers showed that older teachers [3,8,35] and teachers with more experience [12] experience less burnout, but other scholars have found that older teachers are more exhausted and report less engagement [29]. Further, it has been reported that the age variable is not statistically significant at least when the variable is continuous [13]. However, it is necessary to interpret the results within the specific setting. As our sample refers exclusively to the tutor collective and distance university, the evidence may be different in other educational stages and contexts.

Table 8 reveals that all burnout dimensions were worse in tutors that needed to move to another location to teach. The t-test showed statistically significant differences in the DP score (*p* < 0.05) and marginal differences in the EE scores (*p* < 0.1). They also felt higher levels of isolation and a lesser sense of belonging, but the t-test did not show statistically significant differences. The power test of d-Cohen showed a high effect (d > 0.50) for EE (−0.70) and DP (−0.95) and a middle effect (0.20 < d ≤ 0.50) for PA (0.36), IS (−0.25) and SB (0.25).

Table 9 shows greater burnout scores in part-time tutors (tutors that work at the university and also have other work) compared to tutors that work exclusively as university teachers. The scores of EE and DP were higher and they showed lower levels of PA. The t-test and non-parametric test showed statistically significant differences in the DP and PA score (*p* < 0.05). The evidence also reveals that the IS dimension was higher in part-time tutors compared to full-time tutors. Furthermore, SB was lower in part-time tutors compared to full-time tutors. The power test of d-Cohen showed a high effect (d > 0.50) for DP (−1.62), PA (0.99), and SB (−0.69) and a middle effect (0.20 < *d* ≤ 0.50) for EE (0.30) and IS (0.32).

The evidence suggests that burnout dimensions and feelings of IS are worse in part-time tutors. Part-time tutors deal with the normal functions of the university and also with tasks for additional work. Duties at the university are increasing as well as the responsibilities and the controls. The university monitors tutors using a set of different controls. The evidence indicates that it could be difficult to deal with multitasking.

Table 10 reveals that all burnout dimensions were worse when the weight of salary received by the tutoring over the total income was high. The IS and SB dimensions were also worse when the income dependency from the university was higher because they were more exposed to the university and the problems became more serious. The t-test showed statistically significant differences in the EE score (*p* < 0.05) and the non-parametric test showed marginal differences in the EE scores (*p* < 0.1). The power test of d-Cohen showed a high effect (d > 0.50) for EE (−1.04) and SB (0.71) and a middle effect (0.20 < *d* ≤ 0.50) for DP (−0.26), PA (0.49), and IS (−0.20).

Arquero and Donoso [9] suggested that teacher burnout may be related to economic pressure. Leitão et al. [54] found that burnout dimensions are associated with employees’ quality of work life, such as appropriate salary, occupational health, and safe work, among others. Therefore, the burnout de-motivator significantly moderates the relationship between employees’ quality of work life and their contribution to productivity.

## 5. Discussion, Recommendations, Limitations, and Suggestions for Future Research

### 5.1. Discussion

Online learning is gaining momentum in the academic community, businesses, and among policymakers. The acceleration of digitalization and the growth of online education around the world has gradually changed the traditional approach to learning-teaching. This paper examined several stressor factors associated with the teacher profession, such as burnout syndrome and feelings of isolation, in conjunction with the sense of belonging in e-learning and b-learning modalities before and after COVID-19 disruption.

Teacher burnout in online higher education is an interesting topic not only under the umbrella of the pandemic, but also in a normal situation. Hence, several scholars have pointed out that teaching is a stressful profession [4,24] and the levels of teacher burnout are not trivial. Considering that teacher burnout and feelings of isolation can lead to demotivation, negative feelings, dissatisfaction, extremely low levels of efficiency, and low levels of optimism [4], with severe consequences on the physical and mental health of teachers [23,24], teacher burnout is a matter of concern.

This study responds to recent calls in the literature and the ongoing growth of online education around the world. Several reasons motivated the current study: (1) the importance of teacher stress not only for the education context but also for the whole society; (2) the calls for more research about the effects of COVID-19 on society, including emotional, mental, physical, and psychological problems; (3) another critical matter is the scarce attention paid to teacher burnout in e-learning and b-learning modalities compared to conventional education; and (4) another special characteristic of this study is the focus on tutor collective, which are key participants in both e-learning and b-learning modalities. Indeed, the tutor collective has been scarcely examined in the previous literature, and (5) in addition, the discipline is also important because little evidence exists on the analysis of professional stressor factors linked to the work market in accounting courses compared to other disciplines.

The results of this study provide relevant information to expand the literature in the field of health and teaching professionalization. The evidence suggests that tutor burnout is not high, neither before COVID-19 disruption nor after COVID-19 disruption. Despite the score dimensions being slightly worse after the COVID-19 disruption, the statistical tests did not show significant differences between both periods. The results can be explained by the context. Distance universities have extensive experience in dealing with the online setting, supporting a social presence, teaching presence, and cognitive presence in learning. Although all instructors have had to change some procedures, the high support of the institution in our distance education model during this special situation has prevented significant increases in the levels of teacher stress. Therefore, the disruption caused by COVID-19 has not significantly increased burnout symptoms as it has been noted in another context and for other educational models.

The results also revealed that feelings of isolation were not high in the tutor collective. This result is relevant as previous research in the field suggests that feelings of isolation are an intrinsic characteristic of online settings, affecting all the participants and collectives. Our evidence shows that feelings of isolation did not increase after the COVID-19 disruption. The statistical tests did not show significant differences between both periods. The high flexibility and adaptation of online education to cope with changes and specific situations, especially the e-learning modality, can explain why the feelings of isolation in both periods were similar. Another interesting result is the sense of belonging to the teaching community. Our evidence suggests that the tutor collective experienced a high sense of belonging. This is a relevant result for distance universities as this collective differs from the role of regular teachers. It is important to remark that generally distance universities provide great support to this collective.

The sociodemographic and labour variables also showed interesting results. Considering the teaching modality, tutors involved only in the online learning-teaching process (asynchronous modality) felt less exhausted compared to tutors involved in the online modality but with face-to-face classes (synchronous modality). However, the former felt greater levels of depersonalization and low levels of personal accomplishment compared to the latter. Although we found variations in the dimension scores between both modalities, the statistical tests were not significant and the power test showed a middle effect.

The results also showed that EE, PA, and IS, were higher in older and more experienced tutors compared to less mature and less experienced tutors. Although most previous research has found that younger teachers with less experience have greater burnout levels than older or more experienced teachers, the specific setting of this paper makes an additional interpretation necessary. Our results could be interpreted by the technological gap, which is generally more evident for older tutors than for younger tutors. Given that teaching requires online support in both the e-learning and b-learning modalities, younger tutors generally show faster and better adaptation to technological change. This is another interesting result.

Burnout, isolation, and sense of belonging were worse for tutors teaching out of their family location, as well as for part-time tutors. Family location showed statistically significant differences in DP and marginal differences in the EE score. The power test measured by d-Cohen revealed high effects for EE and DP. The evidence also revealed worse levels of the IS and SB dimensions when tutors taught in another locality. This means that this is a relevant variable in distance universities. Indeed, the UNED has more than 60 campuses in the country and outside. It is common to move to another location to teach face to face. Thus, the institution needs to consider this issue to minimize and prevent burnout symptoms.

The evidence also showed interesting results according to work time devoted to university teaching. Burnout and feelings of isolation were higher in part-time tutors, while the sense of belonging was lower in part-time tutors. Although the statistics should be interpreted with caution, we found that the differences were statistically significant in DP and PA with high statistical power. We believe that the university should pay more attention to this group of part-time tutors to increase the levels of PA and SB and decrease the levels of EE, DP, and IS. Hence, most tutors are part-time, constituting an important piece in the online modality. The burnout dimensions were worse when the weight of the salary received from tutoring over the total income was high. This is logical because this group of tutors are very interested in keeping their work at the university and the pressure increases. We found statistically significant differences in the EE score with high statistical power. Although the statistical tests are difficult to interpret due to the sample size, this study takes another step forward from the existing studies.

Taken together, we think that the results of the study make progress in the field and could help distance universities not only to deal with teacher stress but also to minimize and prevent it. The consequences of burnout, feelings of isolation, and sense of belonging will be permanent in universities, with several negative effects in students and the whole society, unless actions and measures are taken. The results of this study can be valuable for future planning and designs in the tutor collective in distance universities.

### 5.2. Recommendations

E-learning is characterized by complexities, such as an asynchronous learning environment, rapid technology changes, time management, perceptions of isolation, learners’ diversity, and multicultural factors, among others. The different context of e-learning and b-blended teaching compared to contemporaneous education, and the expected growth of these two modalities in the coming years for all institutions allow us to provide some recommendations. Indeed, the possibilities that e-learning and b-learning is offered for continuous trainers, demanded by the labour market, as well as the flexibility to cope with future pandemics make it necessary to take a step forward.

Online education is highly exposed to technology changes, which generate new working conditions in the daily teaching work. Whether teachers are not correctly trained to deal with technology (because of low-level preparedness, no knowledge of the didactic possibilities, resistance to using it, feelings of a lack of support to implement it in the classroom, feelings of incompetency, among others), it can result in stress factors, such as burnout and anxiety (see the literature reviewed provided by [1]). Better trained teachers are able to deal with emergency situations, such as the COVID-19 crisis, but are also able to mitigate loneliness and other stress factors like a lack of support, burnout, and socialization. Aside from the COVID-19 outbreak, how the faculty deals with the new education context, where digitalization and innovation play a fundamental role, is a challenge for future papers looking to contribute to teaching quality and implications for mental health.

Special attention is also required for teacher burnout prevention. Teachers need to make efforts to deal with potential stressor variables. In this sense, self-regulation strategies, co-regulation, collaboration with peers, and proactive strategies can help to cope with stress. Universities also need to make progress in the organizational and environmental area. They need to be conscious that teachers’ stress is a serious problem for society. Consequently, it is extremely important to invest in burnout prevention. Undoubtedly, an ideal solution would be to train teachers to afront themselves coming into stressor situations, but some support is necessary. Then, teachers’ learning and continuous community support would be ideal to train future professionals in a complex world.

From our point of view, there are some crucial factors to continue improving the system: the instructor training (teachers and tutors), the teaching motivation, and also strengthing the learning communities for teachers, not only within institutions but also among institutions. Only with adequate collaboration between all the agents can the system improve, with effects on all of society.

### 5.3. Limitations and Suggestions for Future Research

The main limitation of the study is the sample size. Although the response rate was 55% and more than 1120 data were treated, the absolute number of tutors involved in the analysis did not permit other inferential analysis. The results of the statistical tests should be interpreted with caution due to the small sample size. Although we used several statistical tests and power tests, the evidence cannot be extrapolated to the entire academic population, and the context must be considered. However, the importance of examining the tutor collective, which in our study taught more than 1300 students distributed worldwide, justifies the need and value of the current work, which is a good starting point for future analyses.

Future works should involve more participants to analyse tutor burnout in this special collective. It would be also interesting to increase the number of years and courses, including other disciplines, to obtain a deeper understanding of tutor burnout. Another avenue is to combine quantitative analysis with qualitative analysis in order to understand the teacher burnout phenomenon and the interrelation among different factors better. Finally, more research is need in b-learning and e-learning education, including all the agents involved in these education modalities.

## 6. Conclusions

The main results of this study did not reveal high levels of teacher burnout and feelings of isolation, while the sense of belonging was high. The evidence suggests that the disruptive effect of COVID-19 was mitigated by the system itself. Specifically, the high flexibility and adaptation provided by online education was able to cope with the COVID-19 disruption, perhaps in a better way than conventional education, where, in general, scholars have shown an increase in mental, physical, and psychological symptoms. In the online setting, the daily routine of instructors (teachers and tutors) means the virtual environment is not seen as “an enemy”. Indeed, b-tutors who have performed a mandatory switch from face-to-face classes to online classes did not experience high levels of burnout and isolation. In the online setting, the virtual environment is a meeting point that allows interaction with the learning-teaching community and socialization. The networking, interactivity with peers, and the social presence in the virtual environmental may have mitigated some negative effects and mental health problems associated with the pandemic.

The pedagogical models, dynamic methodology, and innovative resources provided by e-learning and hybrid models make the system attractive by itself. Hence, distance education offers an umbrella of possibilities for lifelong education or continuous learning for all the population, supporting a social mission, which contributes positively to the growth of human beings and the continuous training demanded by the labour market. It is important to reflect on the social and health functions that training provides for the growth of human beings and the fulfilment of personal expectations. Distance education also supports the inclusive education promoted by the 2030 Agenda for Sustainable Development issued by United Nations Member States.

In this setting, we can ask ourselves whether the pandemic will involve a change in the education paradigm. The future also holds new challenges for e-learning education models due to the unstoppable digitization, which will include new attractive learning tools, innovative materials, and pedagogical models in the coming years.

## Figures and Tables

**Table 1:** Sample descriptive statistics.

Variables	Type	Weight
Gender	Female	35.71
	Male	64.29
Tutoring modality	Face-to-face with AVIP class	75.00
	Intercampus (AVIP class)	25.00
Age	20–25	7.14
	26–35	21.43
	36–45	46.43
	46–55	25.00
Tutorial tenure	0–5	35.71
	6–10	7.14
	11–20	32.14
	+20	25.00
Teaching location	Family location	57.14
	Out of the family location	42.86
Family responsibilities	Yes	82.14
	No	17.86
Work time devoted to university teaching	Part-time	75.00
	Full-time	25.00
Weight of income by tutorial function	Less than 20%	76.92
	21%–40%	15.38
	More than 80%	7.69

**Table 2:** MBI, IS, and SB dimensions.

	*n*	Number of Items	Mean	SD
EE	28	9	13.75	3.91
DP	28	5	7.96	2.43
PA	28	8	31.89	3.97
IS	28	9	19.57	5.83
SB	28	4	15.96	2.63

**Table 3:** Correlation among dimensions.

	Burnout	IS	SB
Burnout	1	0.346	−0.089
		(0.071)	(0.654)
IS	0.346	1	−0.392
	(0.071)		(0.039)
SB	−0.089	−0.392	1
	(0.654)	(0.039)	

**Table 4:** Results of the dimensions before and after COVID-19.

	Pre-COVID-19			Post-COVID-19		
	*n*	Mean	SD	*n*	Mean	SD
EE	12	13.42	3.75	16	14.00	4.13
DP	12	7.58	2.54	16	8.25	2.38
PA	12	32.33	4.08	16	31.56	3.98
IS	12	20.25	6.30	16	19.06	5.62
SB	12	16.58	2.54	16	15.50	2.68

**Table 5:** Results of the dimensions by tutoring modality.

	Traditional Tutors			Intercampus Tutors		
	(Face-to-Face + AVIP Class by Platform)			(Only AVIP Class by Platform)		
		B-Learning			E-Learning	
	*n*	Mean	SD	*n*	Mean	SD
EE	21	14.19	4.06	7	12.43	3.36
DP	21	7.67	2.08	7	8.86	3.29
PA	21	32.19	4.48	7	31.00	1.63
IS	21	19.76	5.12	7	19.00	8.08
SB	21	15.71	2.85	7	16.71	1.80

**Table 6:** Results of the dimensions by age.

	25–35			36–45			46–55			56–65		
	*n*	Mean	SD	*n*	Mean	SD	*n*	Mean	SD	*N*	Mean	SD
EE	2	11.50	0.71	6	12.00	3.10	13	13.54	4.58	7	16.29	2.56
DP	2	8.50	3.54	6	7.83	2.71	13	8.15	2.34	7	7.57	2.64
PA	2	33.00	2.83	6	31.00	1.55	13	33.62	4.50	7	29.14	3.24
IS	2	17.50	6.36	6	19.50	8.26	13	18.77	5.99	7	21.71	3.09
SB	2	17.00	1.41	6	16.00	1.26	13	16.62	3.18	7	14.43	2.30

**Table 7:** Results of the dimensions by tutorial tenure.

	0–5			6–20			+20		
	*n*	Mean	SD	*n*	Mean	SD	*n*	Mean	SD
EE	10	12.10	2.38	11	13.91	5.09	7	15.86	2.73
DP	10	9.00	2.36	11	7.36	2.54	7	7.43	2.15
PA	10	32.50	4.35	11	32.55	3.33	7	30.00	4.28
IS	10	20.30	6.72	11	16.73	4.90	7	23.00	4.00
SB	10	17.40	2.12	11	15.45	2.34	7	14.71	3.09

**Table 8:** Results of the dimensions by teaching location.

	In the Family Location			Out of the Family Location		
	*n*	Mean	SD	*n*	Mean	SD
EE	16	12.63	3.42	12	15.25	4.16
DP	16	7.06	2.26	12	9.17	2.17
PA	16	32.50	4.24	12	31.08	3.58
IS	16	18.94	6.18	12	20.42	5.48
SB	16	16.25	2.86	12	15.58	2.35

**Table 9:** Results of the dimensions by work time devoted to university teaching.

	Part-Time			Full-Time		
	*n*	Mean	SD	*n*	Mean	SD
EE	21	14.05	4.22	7	12.86	2.85
DP	21	8.52	2.52	7	6.29	0.95
PA	21	30.57	2.84	7	35.86	4.41
IS	21	20.05	6.06	7	18.14	5.24
SB	21	15.52	2.50	7	17.29	2.75

**Table 10:** Results of the MBI dimensions by weight of income by tutorial function.

	Less than 20%			More than 20%		
	*n*	Mean	SD	*n*	Mean	SD
EE	20	12.75	3.35	6	16.67	5.01
DP	20	8.05	2.35	6	8.67	2.58
PA	20	32.35	4.56	6	30.33	1.51
IS	20	19.25	6.39	6	20.50	5.21
SB	20	16.40	2.70	6	14.50	2.51

## Data Availability

The data are not publicly available due to participants’ privacy.
